# Supplementary figures and images for: Are dogs with congenital hearing and/or vision impairments so different from sensory normal dogs? A survey of demographics, morphology, health, behaviour, communication, and activities
Source: PLoS One. 2020 Sep 4;15(9):e0230651. doi: 10.1371/journal.pone.0230651 (PMC7473589; doi:10.1371/journal.pone.0230651)

## HNVI

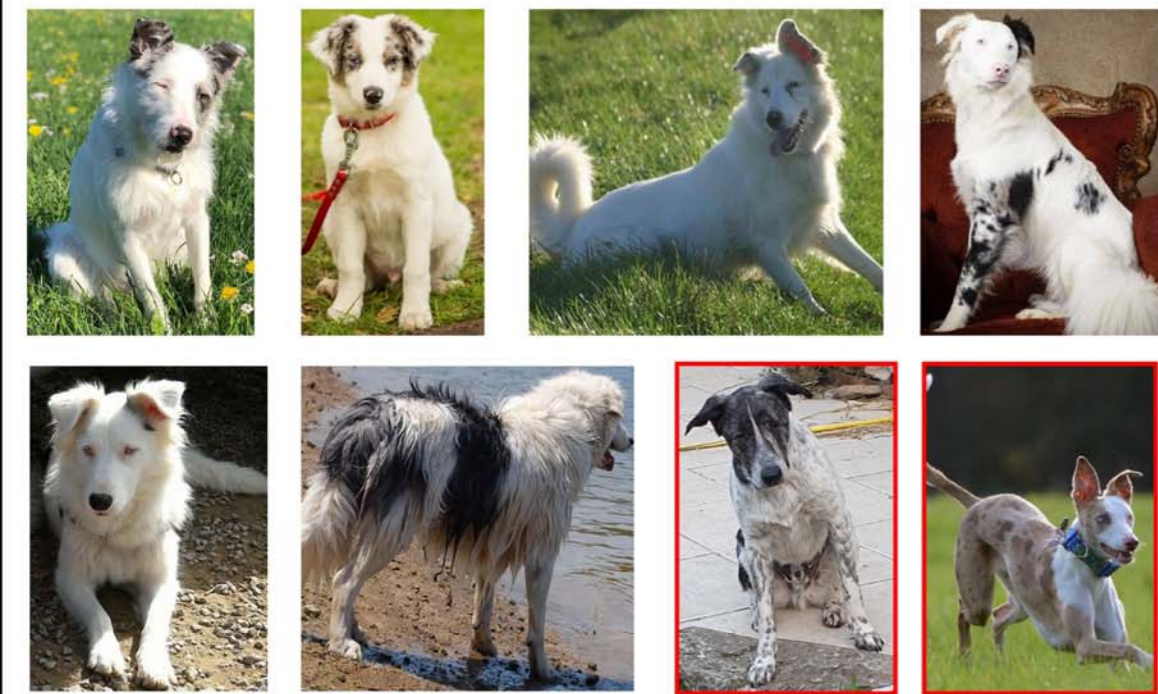

## HIVN

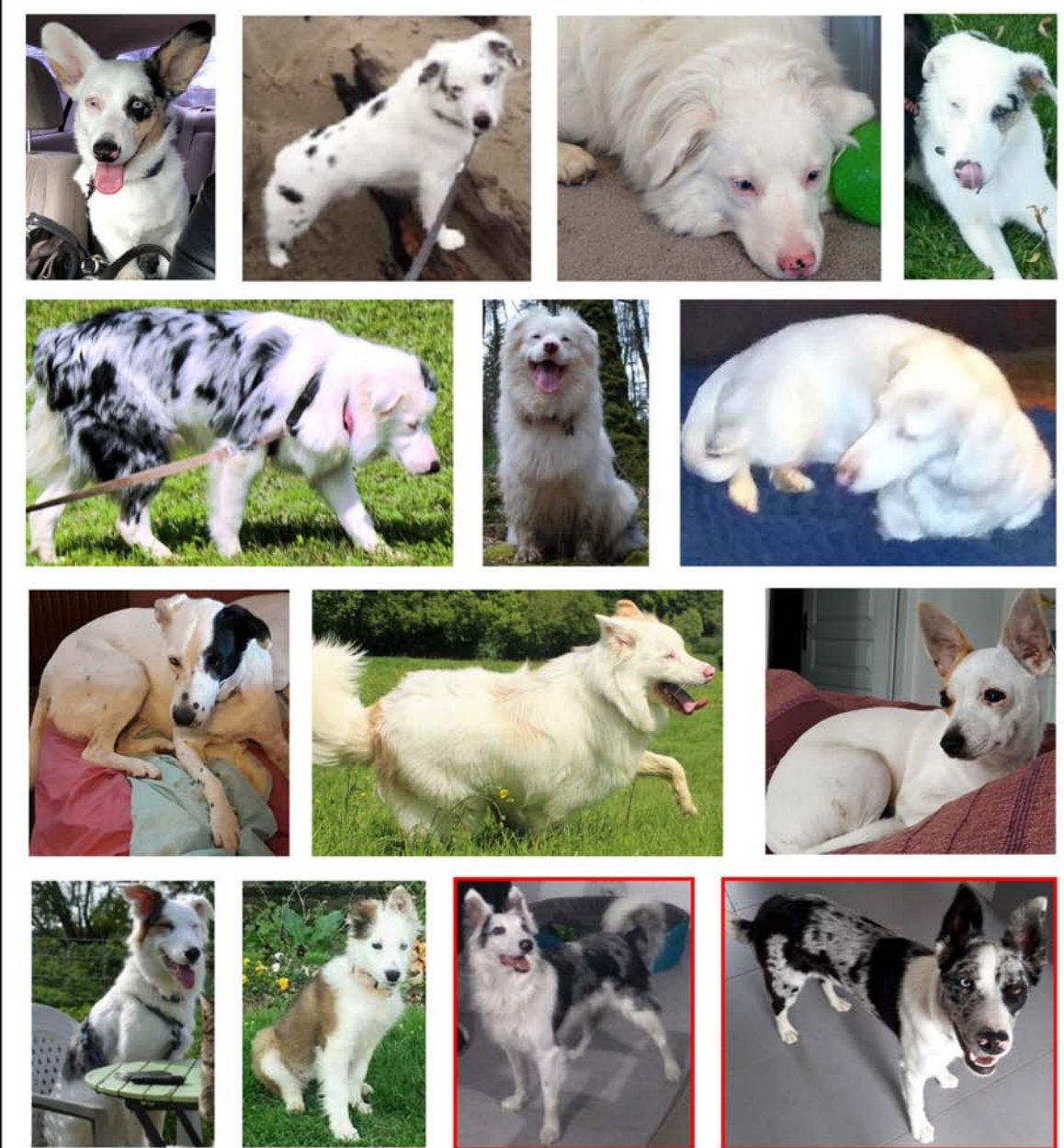

## HIVI

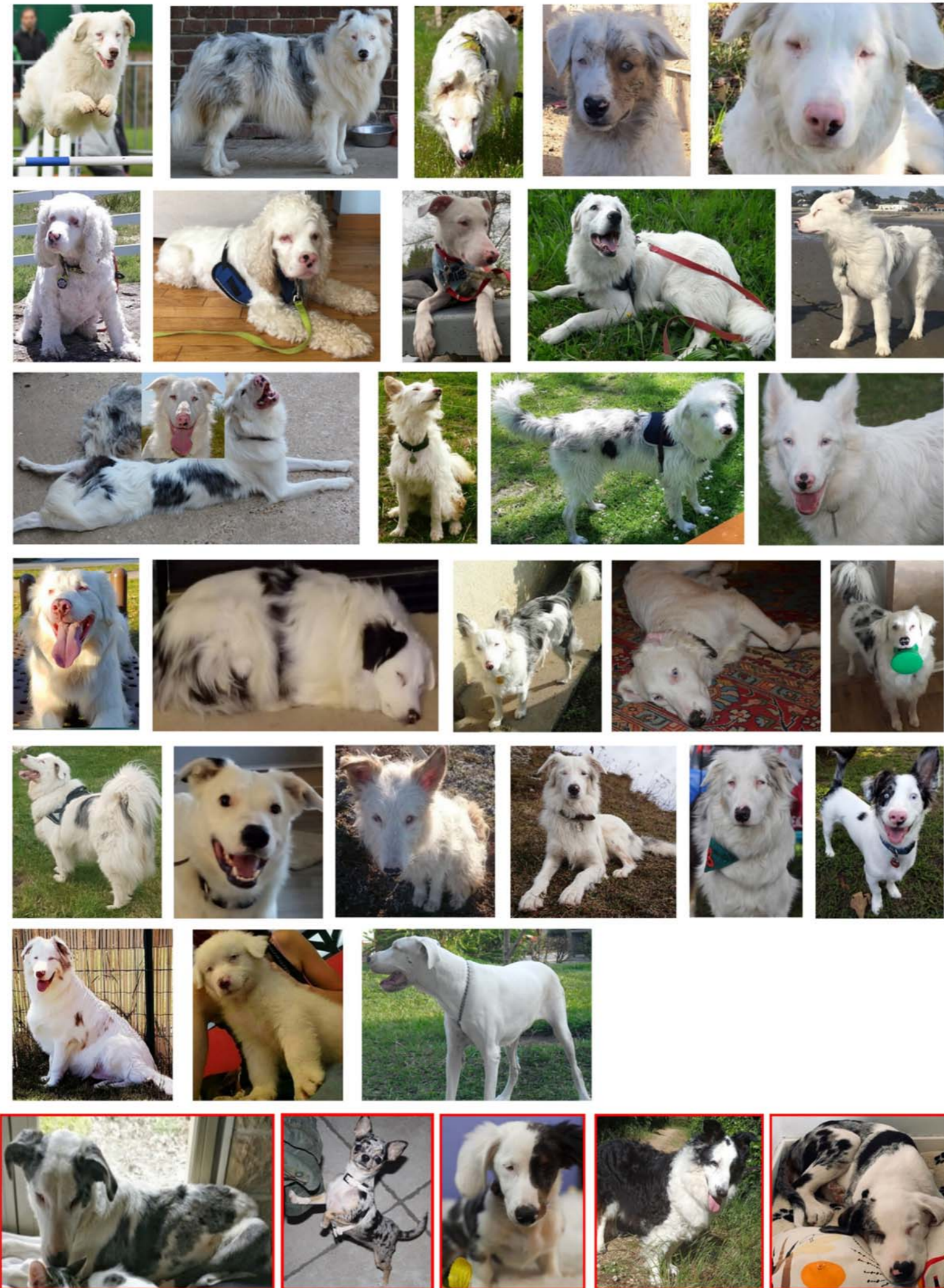

## HNVN

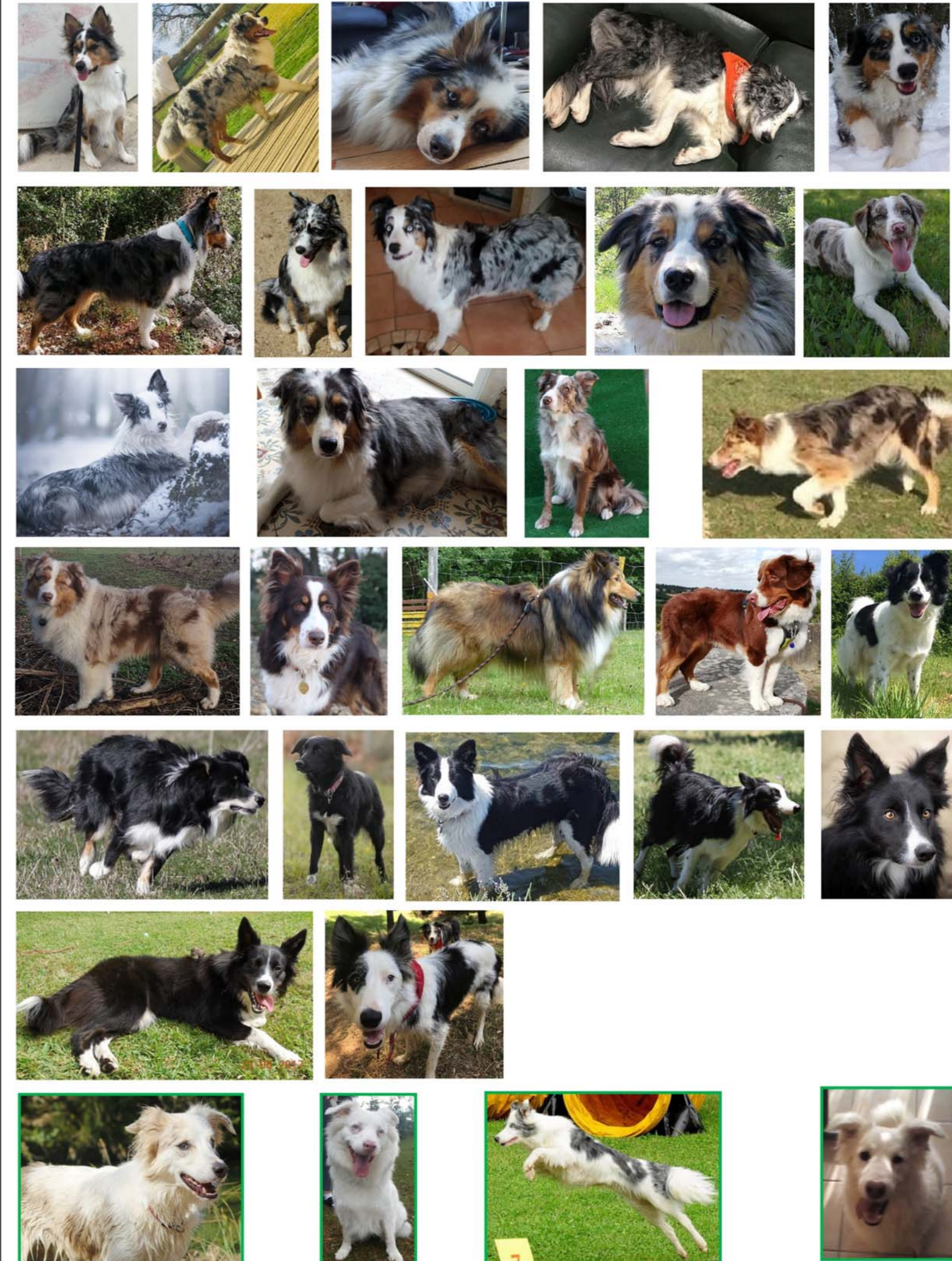

Supplement: S1 Fig — Pictures are sorted by group (HNVI, HIVN, HIVI, HNVN). Pictures of sensory impaired dogs with lesser white in the coat are framed in red. Pictures of sensory normal dogs with excess white coat are framed in green. Each individual picture has been sent by one dog owner to the first author on a voluntary basis. The portfolio has then been created by the first author for this paper. (PDF) [file pone.0230651.s001.pdf]

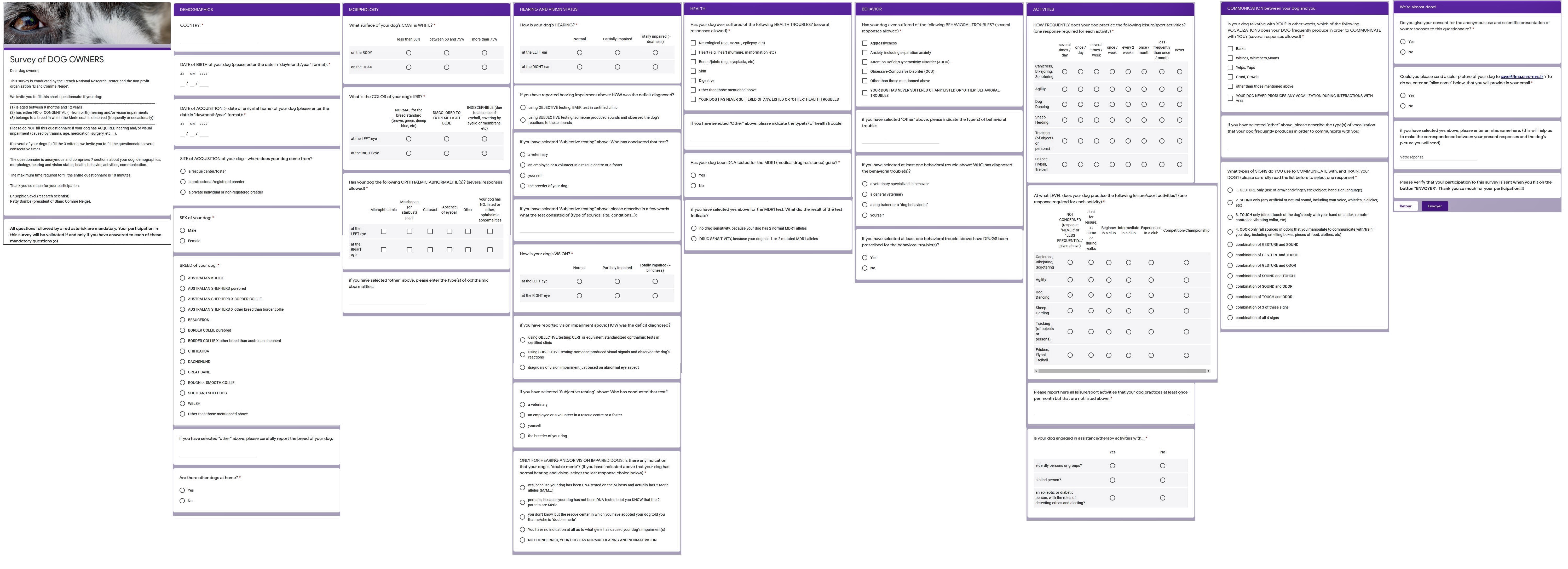

Supplement: S2 Fig — (JPG) [file pone.0230651.s002.jpg]
